# Reelin-Related Disturbances in Depression: Implications for Translational Studies

**DOI:** 10.3389/fncel.2016.00048

**Published:** 2016-02-25

**Authors:** Hector J. Caruncho, Kyle Brymer, Raquel Romay-Tallón, Milann A. Mitchell, Tania Rivera-Baltanás, Justin Botterill, Jose M. Olivares, Lisa E. Kalynchuk

**Affiliations:** ^1^Neuroscience Cluster, College of Pharmacy and Nutrition, University of SaskatchewanSaskatoon, SK, Canada; ^2^Department of Psychology, University of SaskatchewanSaskatoon, SK, Canada; ^3^Department of Medicine, University of SaskatchewanSaskatoon, SK, Canada; ^4^Department of Psychiatry, Alvaro Cunqueiro Hospital, Biomedical Research Institute of VigoGalicia, Spain

**Keywords:** reelin, depression, hippocampus, neurogenesis, neural plasticity, lymphocytes, membrane protein clustering

## Abstract

The finding that reelin expression is significantly decreased in mood and psychotic disorders, together with evidence that reelin can regulate key aspects of hippocampal plasticity in the adult brain, brought our research group and others to study the possible role of reelin in the pathogenesis of depression. This review describes recent progress on this topic using an animal model of depression that makes use of repeated corticosterone (CORT) injections. This methodology produces depression-like symptoms in both rats and mice that are reversed by antidepressant treatment. We have reported that CORT causes a decrease in the number of reelin-immunopositive cells in the dentate gyrus subgranular zone (SGZ), where adult hippocampal neurogenesis takes place; that down-regulation of the number of reelin-positive cells closely parallels the development of a depression-like phenotype during repeated CORT treatment; that reelin downregulation alters the co-expression of reelin with neuronal nitric oxide synthase (nNOS); that deficits in reelin might also create imbalances in glutamatergic and GABAergic circuits within the hippocampus and other limbic structures; and that co-treatment with antidepressant drugs prevents both reelin deficits and the development of a depression-like phenotype. We also observed alterations in the pattern of membrane protein clustering in peripheral lymphocytes in animals with low levels of reelin. Importantly, we found parallel changes in membrane protein clustering in depression patients, which differentiated two subpopulations of naïve depression patients that showed a different therapeutic response to antidepressant treatment. Here, we review these findings and develop the hypothesis that restoring reelin-related function could represent a novel approach for antidepressant therapies.

## Brief History of Reelin in the Nervous System

The reeler mouse has been widely studied since the 1950’s as a model to understand neural development and developmental dysregulations (reviewed in Lambert de Rouvroit and Goffinet, 1998). However, when the reelin gene was cloned by D’Arcangelo et al. (1995), a new field focused on the neurobiology of reelin was firmly established. Early on, reelin was primarily thought of as a developmental molecule highly expressed in cortical and hippocampal Cajal-Retzius cells and cerebellar granule cells (see Tissir and Goffinet, 2003), but very soon came the demonstration that reelin is preferentially expressed by GABAergic interneurons in the adult cortex and hippocampus of rodents (Alcantara et al., 1998; Pesold et al., 1998, 1999). This was subsequently shown in many other species, including non-human primates and humans (Martínez-Cerdeño et al., 2002; Rodriguez et al., 2002; Roberts et al., 2005; Ramos-Moreno et al., 2006). Although studies with reeler mice already indicated that reelin played an important role in regulating neural migration during brain development (see Lambert de Rouvroit and Goffinet, 1998), additional data demonstrated key roles for reelin in dendritic maturation and dendritic spine development (Niu et al., 2008; Chameau et al., 2009), in promoting synaptic plasticity in spine-impinging synapses, and in memory formation in the adult brain (Pesold et al., 1999; Rodriguez et al., 2000; Weeber et al., 2002; Beffert et al., 2005, 2006; Pujadas et al., 2010). More recently, it has become clear that reelin influences various aspects of hippocampal neurogenesis, including neural progenitor fate, neuronal migration, dendritic spine development and the integration of granule neurons into hippocampal circuitry. The absence of Dab1, a protein that is part of the reelin signal transduction pathway, limits dendritic development in dentate neuroprogenitor cells and causes those cells to migrate ectopically into the hilus (Teixeira et al., 2012). However, enhancing hippocampal reelin levels seems to normalize migration and increase the maturation rate of newborn granule neurons (Pujadas et al., 2010; Teixeira et al., 2011). Taken together, these results outline an important role for reelin in regulating hippocampal plasticity in the adult brain.

Although the vast majority of research on reelin has been conducted in the mammalian brain, reelin is also present in non-mammalian species. For example, reelin is highly expressed in the larval sea lamprey brain, particularly during the metamorphic stage of development (Pérez-Costas et al., 2002, 2004). Lampreys are primitive vertebrates with a laminar brain and no conventional radial-migration during development. Given that reelin is a key regulator of radial-migration in the rodent brain (Rakic and Caviness, 1995), the presence of reelin in larval sea lampreys suggests that in phylogenetic terms, reelin could have initially evolved as a molecule for regulating synaptic remodeling, and only later on became important for neural migration (Pérez-Costas et al., 2002).

The conceptualization of reelin as a pleiotropic extracellular matrix molecule with multiple roles in brain development and in adult brain plasticity captured the attention of Erminio Costa and Alessandro Guidotti, who hypothesized that reelin expression could be dysregulated in psychotic disorders (i.e., schizophrenia), and that baseline levels of reelin could be an important vulnerability factor in a two-hit neurodevelopmental hypothesis for the development of schizophrenia. Their initial studies revealed a downregulation of about 50% of brain reelin expression levels in both schizophrenia and bipolar disorder (Impagnatiello et al., 1998; Guidotti et al., 2000). These results were independently replicated by several research groups (Fatemi et al., 2000; Eastwood and Harrison, 2003; Knable et al., 2004; Torrey et al., 2005; Habl et al., 2012), opening the field for subsequent investigation of how reelin dysregulation might be operative in the pathogenesis and/or pathophysiology of multiple psychiatric disorders. Years later, Costa and Guidotti pioneered the study of epigenetic alterations as the possible cause of reelin downregulation in psychotic disorders (Veldic et al., 2004; recently reviewed in Grayson and Guidotti, 2013; Guidotti and Grayson, 2014).

It was Hossein Fatemi who first suggested that reelin may be downregulated in autism spectrum disorders (Fatemi et al., 2001b, 2002, 2005; Fatemi, 2002, 2005a,b). This led to the idea of a co-occurrence of reelin disturbances in autism and schizophrenia (Fatemi, 2010; Folsom and Fatemi, 2013). In addition, Fatemi’s group also showed that a downregulation of reelin in the hippocampus occurred not only in schizophrenia and bipolar disorder, but also in patients with depression. In this case, they described a large but non-significant reduction of reelin-positive cells in the hippocampal CA4 region (the polymorphic region of the dentate gyrus; Fatemi et al., 2000).

The observation that hippocampal reelin is decreased in patients with depression led our research group to conduct a series of experiments to systematically examine whether reelin is altered in an animal model of depression. Beyond the patient data described above, it seemed to us that both chronic stress (an important risk factor for depression) and deficient reelin produce strikingly similar alterations in hippocampal plasticity and function. One example if this is the fact that both stress and deficient reelin can impair adult hippocampal neurogenesis and the proper maturation and integration of newborn neurons in the dentate gyrus (Pujadas et al., 2010; Lussier et al., 2013a), which have been repeatedly implicated in the pathogenesis of depression (as a recent review, see Schoenfeld and Cameron, 2015). The remainder of this review describes our studies of reelin-related events in the hippocampus and periphery of rats using a well characterized animal model of depression (Kalynchuk et al., 2004; Gregus et al., 2005; Johnson et al., 2006; Marks et al., 2009; Sterner and Kalynchuk, 2010), as well as our examination of peripheral biomarkers in depression patients (Rivera-Baltanás et al., 2012, 2014, 2015). We conclude by offering some hypotheses about how these ideas could be explored in a translational way in relation to modifying the reelin system to develop better diagnoses, prognoses, and therapeutics for patients with major depression.

## Reelin Expression in the Hippocampus: Putative Role in Depression

We first assessed possible deficits in the number of reelin-immunopositive cells throughout the hippocampus in two chronic stress paradigms. Many current animal models of depression are based on chronic or repeated stress paradigms, because there is a well established relationship between exposure to traumatic or stressful life events and the onset of depressive symptoms in patients (Keller et al., 2007). In this case, we used a repeated corticosterone (CORT) injection paradigm, in which rats receive a 40 mg/kg injection of CORT once per day for 21 consecutive days, and a repeated restraint stress paradigm, in which rats are placed in plastic restraint tubes for 6 h/day for 21 consecutive days. We chose these two stress paradigms because we and others had previously found that repeated CORT injections reliably increase depression-like behavior but repeated restraint stress does not (Gregus et al., 2005; Brummelte and Galea, 2010; Workmann et al., 2013; Kott et al., 2016). If alterations in hippocampal reelin are involved in depression, we would expect to see a loss of reelin-positive cells in rats subjected to the CORT injections but not restraint stress. Our results were consistent with this hypothesis, as we found a significant decrease (26%) in the number of reelin-positive cells specifically in the subgranular zone (SGZ) of the dentate gyrus after repeated CORT injections but not repeated restraint stress (Lussier et al., 2009). The fact that reelin-positive cells were lost in the SGZ was notable, as this is the location where adult hippocampal neurogenesis takes place and from where newborn neurons migrate into the granular cell layer and develop into mature granule cells, with dendrites extending through the dentate molecular layer and axons projecting toward CA3 pyramidal cells. This suggested to us that stress-induced alterations in reelin-expressing GABAergic interneurons located adjacent to the SGZ could influence the course of hippocampal neurogenesis. To investigate this idea, we compared the time course of changes in the number of reelin-positive cells in the SGZ, the maturation rate of newborn granule neurons, and the onset of a depression-like phenotype in rats subjected to 7, 14, or 21 days of CORT injections. We found that CORT-treated rats showed gradual increases in depression-like behavior over the course of the injections, which were paralleled by significant decreases in SGZ reelin expression (no changes at 7 days, 25% decrease at 14 days, and 26% decrease at 21 days) and significant decreases in the number of surviving immature dentate granule cells and the complexity of dendritic processes present in surviving immature granule cells (Lussier et al., 2013a). We interpreted these observations to indicate that reelin downregulation may delay the maturation of newborn granule cells and impair proper integration of these neurons into mature circuits, thereby disrupting hippocampal circuitry and enhancing depression-like behavior. This conclusion is consistent with other findings that reeler mice (with null reelin expression) have fewer mitotic cells in the dentate SGZ than wildtype mice (Sibbe et al., 2015) and that inactivation of the reelin pathway impairs hippocampal adult neurogenesis (Teixeira et al., 2012). It also adds a new component to the prominent hypothesis that adult hippocampal neurogenesis plays a causal role in major depression (Jacobs et al., 2000). This hypothesis was derived from data showing that patients with depression have reduced hippocampal volume (MacQueen et al., 2003; Campbell et al., 2004) and that chronic stress and antidepressant drugs can decrease and increase cell proliferation and survival respectively (Santarelli et al., 2003; Petrik et al., 2012). However, the putative causal role of neurogenesis in depression has been controversial because in animal models, both depression-like behavior and the behavioral actions of antidepressants can be dissociated from alterations in hippocampal neurogenesis (Surget et al., 2008; Bessa et al., 2009; David et al., 2009). Additionally, close examination of postmortem tissue from depressed patients has not revealed significant decreases in hippocampal stem cell proliferation (Lucassen et al., 2001; Reif et al., 2006). Our reelin data point to the idea that depressive symptoms could be associated with deficient neuronal maturation and integration rather than cell proliferation and survival *per se*. This idea has also been suggested by other research groups (Bessa et al., 2009; Mateus-Pinheiro et al., 2013).

Although many studies of reelin in animal models of depression have focused on the dentate SGZ and adult hippocampal neurogenesis, this is not the only region of the hippocampus where reelin is altered after a period of chronic stress. We have also reported that repeated CORT injections significantly decrease the number of reelin-positive cells in the CA1 stratum lacunosum-moleculare (by 21%), and dampen the co-expression of reelin and neuronal nitric oxide synthase (nNOS) in the molecular layer of the dentate gyrus (Lussier et al., 2009; Romay-Tallón et al., 2015). Early descriptions of reelin immunolabeling in the adult hippocampus identified a heavy “diffuse labeling” in both the distal molecular layer of the dentate gyrus and the CA1 stratum lacunosum-moleculare (Pesold et al., 1998), which was interpreted to indicate the presence of reelin secreted into the extracellular matrix that would regulate the strength of synaptic connections onto distal dendritic spines (Pesold et al., 1999; Rodriguez et al., 2000). Later on, it was shown that reelin signaling regulates glutamate receptor composition and activity particularly in the distal dendritic compartment (Chen et al., 2005; Sinagra et al., 2005; Qiu et al., 2006; Groc et al., 2007; Campo et al., 2009; Iafrati et al., 2014; Kupferman et al., 2014), and also that reelin could play a role in neurotransmitter release (Hellwig et al., 2011; Bal et al., 2013). The downregulation and neurochemical alterations of reelin-positive cells in the distal molecular layer and CA1 stratum-lacunosum-moleculare instigated by chronic stress could then result in changes in synaptic strength and/or glutamatergic receptors, and/or neurotransmitter release that will further affect hippocampal circuitry. This fits quite nicely with our report that repeated CORT injections significantly decrease expression of GAD65 and the GABA_A_ α2 receptor subunit in the amygdala and hippocampus and increase expression of VGLUT2 within the hippocampus (Lussier et al., 2013b). These changes would create an imbalance in glutamatergic-GABAergic neurotransmission within the hippocampus, which could be another important pathophysiologic event in depression that is instigated by a deficit in reelin.

Heterozygous reeler mice have been used as a way to study the functional consequences of genetic deficits in reelin expression. These mice have about 50% of normal levels of reelin in bothe the brain and peripheral tissues. Previous work with these mice revealed mild neurochemical (Liu et al., 2001; Pappas et al., 2001; Ballmaier et al., 2002; Isosaka et al., 2006; Romay-Tallón et al., 2010; Nullmeier et al., 2011; Ventrutti et al., 2011; Varela et al., 2015), and behavioral (Tueting et al., 1999, 2006) alterations that did not appear to give rise to an overt pathological phenotype, but that could prime these animals to a high vulnerability to the deleterious effects of chronic stressors. We tested this idea by investigating whether heterozygous reeler mice would be more susceptible to the depressogenic effects of repeated CORT injections than wildtype mice. Groups of heterozygous reeler mice and wildtype mice received daily injections of CORT (i.e., at 5 mg/kg, 10 mg/kg, or 20 mg/kg) over a 21-day period. We found that in the absence of CORT, heterozygous reeler mice do not show more depression-like behavior or deficits in the number or maturation rate of immature neurons compared to wildtype mice. However, the heterozygous reeler mice were more susceptible than wildtype mice to the damaging effects of CORT, as they showed dose dependent increases in depression-like behavior and decreases in neuronal survival and maturation (Lussier et al., 2011). Furthermore, analysis of the colocalization of reelin and nNOS in CORT-treated heterozygous reeler mice revealed a genotype × treatment interaction, with increased colocalization of both markers in the dentate SGZ in CORT treated heterozygous reeler mice. This indicates that chronic stress increases nNOS expression in reelin-positive cells when baseline levels of reelin are relatively low. This increase in nNOS expression could stimulate the release of nitric oxide, giving rise to an excitotoxic event through hyperactivation of NMDA glutamate receptors in these cells. Hypothetically, this excitotoxicity could have downstream consequences such as a loss of reelin release from GABAergic interneurons and the interruption of normal migration and maturation of newborn dentate granule cells (Romay-Tallón et al., 2015), as discussed above.

**Figure 1:**
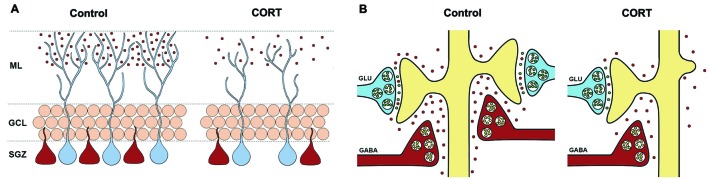
**Graphical depiction of two possible mechanisms by which reelin down-regulation may be involved in the pathophysiology of depression. (A)** Reelin secreted by some hippocampal GABAergic interneurons in the dentate subgranular zone (SGZ) as well as in cells in the distal molecular layer (shown in red) is involved in hippocampal neurogenesis, particularly the rate and extent of dendritogenesis of newborn granule cells (shown in blue). Repeated CORT administration reduces the number of reelin+ cells in the dentate SGZ and the amount of extracellular reelin in the distal molecular layer, which delays the maturation (e.g., reduced dendritogenesis) of newborn neurons. These neurons may fail to properly integrate into existing hippocampal circuits. **(B)** Reelin secreted by GABAergic interneurons promotes and stabilizes synapses impinging onto dendritic spines. The downregulation and neurochemical alterations of reelin-positive cells in the distal molecular layer and CA1 stratum-lacunosum-moleculare (shown in red) instigated by chronic stress would decrease in the number of dendritic spines, resulting in a loss of glutamatergic synaptic strength and possibly a dampening of neurotransmitter release from glutamatergic terminals (shown in blue). This would further affect hippocampal circuitry.

Overall, the evidence gathered to date clearly shows that reelin is important for adult hippocampal plasticity and that exposure to chronic stress or high levels of circulating glucocorticoids dampens reelin activity, with subsequent deficits in neuronal maturation and the development of depression-like behavior. Figure 1 shows two possible roles for reelin in the pathogenesis of depression. It should be pointed out that the experiments that these ideas were developed from have all been conducted using male rodents, and there is some evidence that reelin may be differentially altered in male and female rodents under stress conditions (van den Buuse et al., 2012; Buret and van den Buuse, 2014). Nevertheless, the data described above beg the question of whether rescuing or enhancing hippocampal reelin could have antidepressant effects. We have recently demonstrated that administration of the tricylic antidepressant imipramine during the period of CORT injections prevents the downregulation of hippocampal reelin and rescues the behavioral phenotype (Fenton et al., 2015a). Other researchers have also shown that repeated citalopram administration counteracts the loss of hippocampal reelin after kainic-acid treatment (Jaako et al., 2011). Although these experiments do not provide definitive evidence that an enhancement of reelin is directly responsible for better behavioral outcomes, they are certainly consistent with that idea. There is a need for future studies to examine whether reelin enhancement is part of the mechanism underlying the therapeutic effects of antidepressant drugs. Furthermore, recent evidence has shown that intraventricular reelin infusions facilitate hippocampal-dependent cognition in a mouse model of Angelman syndrome (Hethorn et al., 2015). These authors did not report a specific mechanism by which reelin supplementation could facilitate memory in these mice. The identification of this mechanism is an important next step, which could also inform the development of novel antidepressant drugs or mechanisms of action for antidepressant drug actions (see discussion below).

## Peripheral Reelin in Relation to Psychoneuroimmunology

Soon after the cloning of the reelin gene came the demonstration that reelin expression is not exclusive to the central nervous system, but that it is also expressed in other body regions both during developmental stages and adulthood. These regions were primarily identified as the yolk sac and blood vessels during developmental stages, and throughout life in the kidney, liver, and blood (Ikeda and Terashima, 1997; Smalheiser et al., 2000). Since then, reelin expression has also been shown in lymphatic tissues (Samama and Boehm, 2005; Lutter et al., 2012), platelets (Tseng et al., 2010), the enteric nervous system (Bottner et al., 2014), bone marrow (Chu et al., 2014), and some adult brain endothelial cells (Pérez-Costas et al., 2015). Although many studies have focused on the functional roles of reelin in brain development and the adult nervous system, there is scarce knowledge about the functional role reelin might play in the periphery. The picture so far illustrates reelin as a pleiotropic molecule with diverse functional roles both in the brain and periphery: as such, reelin is known to be released from liver and/or kidney cells into blood plasma (Smalheiser et al., 2000), where it plays a role in regulating erythropoiesis in the bone marrow (Chu et al., 2014), and in hemostasis (Tseng et al., 2010, 2014). Reelin also regulates lymphatic vessel formation (Lutter et al., 2012); and the remodeling of the vascular network in reelin-deficient mice (Lindhorst et al., 2012) together with the expression of reelin in yolk sac and developing blood vessels (Ikeda and Terashima, 1997), has led to the idea that reelin may be involved in blood vessel formation.

After our studies indicating that the addition of recombinant reelin to synaptosomes can increase protein expression (Dong et al., 2003), and that this increase was also accompanied by an augmentation of protein clustering on synaptosomal membranes (Caruncho et al., 2004), we wondered whether reelin could regulate membrane protein clustering along the cell membrane of peripheral blood cells (i.e., lymphocytes). Several observations informed this question, including the fact that reelin is highly expressed in blood plasma (Smalheiser et al., 2000), that plasma reelin is altered in mood and psychotic disorders (Fatemi et al., 2001a), that reelin induces clustering of its own receptors receptors and this is an important event for signaling (Strasser et al., 2004), and that lymphocytes contain reelin receptors whose expression is changed in psychiatric disorders (Suzuki et al., 2008). We hypothesized that a decrease in reelin levels or null reelin expression, as observed in heterozygous or homozygous reeler mice respectively, would alter the clustering of some specific proteins, such as the serotonin transporter, that tend to bunch into lipid rafts. We subsequently observed important alterations in the number and size of serotonin transporter clusters in both hererozygous and homozygous reeler mice, particularly in the latter where most lymphocytes showed a diffuse serotonin transporter immunostaining that made it difficult to identify individual clusters (Rivera-Baltanás et al., 2010). Not surprisingly, heterozygous and homozygous reeler mice have dysregulated secretion of cytokines by lymphocytes and macrophages (Green-Johnson et al., 1995).

It is now commonplace to conceptualize inflammatory events as key components in the pathophysiology of depression (see as reviews Raison et al., 2006; Maes et al., 2009; Miller et al., 2009; Leonard, 2010; Blume et al., 2011; Sperner-Unterweger et al., 2014; Young et al., 2014). In fact, levels of inflammatory cytokines are one of the best characterized biomarkers of depression (see as reviews Mössner et al., 2007; Howren et al., 2009; Li et al., 2011; Lichtblau et al., 2013; Valkanova et al., 2013). Taking into account the just mentioned alterations in immune cells in mice expressing low levels of reelin, as well as the disturbances in reelin immunoreactive cells in the dentate gyrus of both depressive patients (Fatemi et al., 2000) and rats showing depression-like behavior (Lussier et al., 2013a), we examined the pattern of lymphocyte membrane protein clustering in peripheral blood samples taken from rats treated with repeated CORT injections (i.e., showing a depressive-like phenotype) and from patients with depression both before treatment and after 8 weeks of antidepressant drug treatment. The pattern of protein clustering of the serotonin transporter and the serotonin 2A receptor on the cell membrane of lymphocytes from rats treated for 3 weeks with CORT showed a significant increase in cluster size for both markers in comparison with control rats, and also a significant positive correlation between larger membrane protein clusters and more depression-like behavior in the forced-swim test (Fenton et al., 2015b). Studies of lymphocytes from depression patients revealed an increase in the size of both serotonin transporter and serotonin 2A receptor protein clusters along the plasma membrane, similar to the alterations found in the CORT-treated rats. However, an important difference is that the analysis of the pattern of clustering of these markers also allowed us to differentiate two subpopulations of naïve depression patients that showed different therapeutic outcomes after antidepressant treatment (i.e., a subpopulation of naïve depression patients that had a poor response to treatment showed smaller clusters and a high percentage of clusters of 0.05–0.10 μm^2^, whereas a subpopulation of naïve patients with better therapeutic outcomes showed larger clusters and a small percentage of clusters of 0.05–0.10 μm^2^). These observations led us to propose that analyses of membrane protein clustering may be a new approach to identify novel biomarkers of depression, and perhaps of other psychiatric disorders as well (Rivera-Baltanás et al., 2012, 2014). Recently, we demonstrated that alterations in serotonin transporter clustering in lymphocytes in depression also correlate with remittance of anhedonia symptoms after antidepressant treatment (Rivera-Baltanás et al., 2015). This finding is of great interest when considering that anhedonia not only represents a cardinal symptom of depression, but it also is generally considered a symptom that shows a relatively poor response to conventional antidepressant treatment (Spijker et al., 2001; Pizzagalli, 2014). Therefore, being able to identify naïve depression patients that will show good or poor improvement in anhedonia symptoms may be useful in a clinical setting.

**Figure 2:**
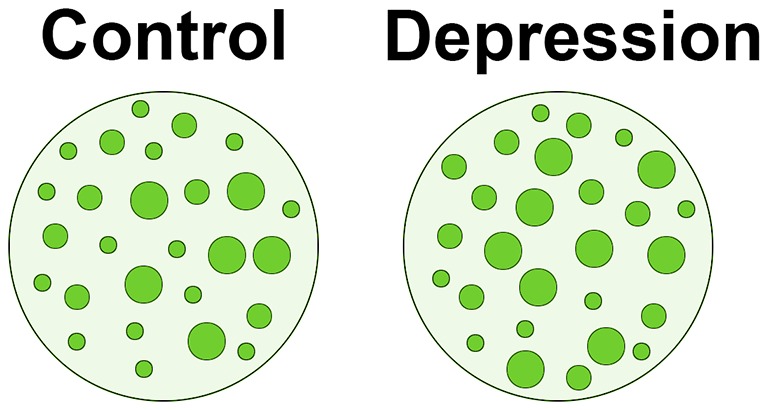
**Schematic representation of alterations in membrane protein clustering in depression.** One of the roles of peripheral reelin may be to regulate membrane protein clustering in lymphocytes (see text for details). If peripheral reelin is decreased in depression, the pattern of protein clustering in peripheral immune cells could be altered. The figure shows our findings that patients with depression have generally larger protein clusters (shown in green) along the lymphocyte plasma membrane than non-depressed subjects (see Rivera-Baltanás et al., 2012, 2014). We propose that alterations in protein clustering in depression patients could stimulate cytokine secretion, as has been frequently reported in depression.

We are currently evaluating if different subpopulations of lymphocytes are differentially affected in terms of membrane protein clustering in depression, and also how these alterations may give rise to changes in the release of cytokines, which is of special interest for us when considering the depressogenic effects of cytokines in anhedonia (Anisman et al., 2002). The hypothesis driving this work is that alterations in reelin expression and membrane protein clustering in peripheral lymphocytes and monocytes may underlie some of the dysregulation in expression of pro-inflammatory and/or anti-inflammatory cytokines that may play a key role in the pathophysiology of major depression. Figure 2 depicts a possible mechanism of how reelin dysregulation may relate to these events.

In relation to reelin expression in plasma and membrane protein clustering in depression, it is also of interest to consider that alterations in the pattern of clustering appear to affect proteins that tend to integrate within lipid rafts, such as the serotonin transporter (Magnani et al., 2004), and that shifting of Gα-proteins to and from lipid raft domains has been postulated as a possible mechanism involved in the chronic effects of antidepressants (Zhang and Rasenick, 2010; Czysz et al., 2015). This opens the possibility of studying combined alterations in membrane protein clustering and Gα-protein translocation to/from lipid rafts as an additional operative mechanism in the pathophysiology of depression that may be amenable to novel therapeutic intervention.

## The Reelin System as a Target for Novel Antidepressants

The idea that reelin could be a part of the mechanism of antidepressant drug action or that it could have beneficial effects on its own was introduced in an earlier section of this review. This idea appears to be gaining momentum in the field: Several investigators have used exogenous reelin (or alternatively, rodent models of reelin overexpression) to investigate if high reelin levels can revert some of the neurochemical, anatomical, functional, and behavioral alterations in various animal models of human pathology. These studies have revealed that reelin supplementation can enhance synaptic plasticity, dendritic spine density, and cognitive ability in wild-type mice (Rogers et al., 2011), and that it can recover synaptic plasticity and learning deficits in heterozygous reeler mice (Rogers et al., 2013). The addition of exogenous reelin also prevents hippocampal dentate granule cell dispersion in experimental epilepsy (Müller et al., 2009), has a preventive effect on phencyclidine-induced behavioral deficits (Ishii et al., 2015), and recovers the neurochemical and behavioral phenotype in a mouse model for Angelman syndrome (Hethorn et al., 2015). Overexpression of reelin also prevents the development of behavioral alterations related to schizophrenia and bipolar disorder (Teixeira et al., 2011), and delays amyloid-beta fibril formation and rescues cognitive deficits in an animal model of Alzheimer’s disease (Pujadas et al., 2014). Overall, these studies paint a tantalizing picture of the possible benefits reelin could have for a number of brain pathologies centered on hippocampal dysfunction. This is of course, also true for the case of major depression. The neurochemical and behavioral deficits associated with a loss of reelin in animal models of depression, together with the rescuing of behavioral phenotypes by addition or overexpression of reelin in models of several neuropsychiatric disorders, strongly suggest that tackling the reelin system (i.e., by the addition of recombinant reelin, by activating the reelin receptors VLDLR and/or ApoER2, or by neuroprotection of reelin-positive cells) could be a good strategy for the development of novel antidepressants.

The studies mentioned above have all used infusions of reelin into the brain. Reelin is a very large protein, and it has been unclear whether peripheral administration of reelin could influence functions within the brain. However, the recent finding of reelin immunoreativity product within caveolar vesicles in endothelial cells in brain regions showing a high level of extracellular reelin labeling suggests that reelin peptides might indeed cross the blood-brain-barrier (Pérez-Costas et al., 2015). This opens the possibility that peripherally administered reelin could influence brain function. It also suggests that alterations in reelin could be important in relation to vascular and/or brain-blood-barrier disturbances in major depression, as these factors appear to be operative in the pathophysiology of depression (Najjar et al., 2013; Taylor et al., 2013).

The pattern of alterations in membrane protein clustering in lymphocytes, together with the multiple observations of disturbances in proinflammatory cytokines in major depression, as discussed in the previous section of this report, also raises the possibility of developing novel therapeutic strategies based on interventions that act peripherally in the immune system. In fact, an interesting recent report has shown that lymphocytes from chronically-stressed mice confer antidepressant-like effects when transferred to naïve mice (Brachman et al., 2015). In translating this remarkable observation to the human condition, one could imagine the possibility of extracting peripheral lymphocytes from patients with treatment-resistant depression and developing conditions to treat them *in vitro* (i.e., conditions that could result in alterations in membrane protein clustering that we have found to relate to a good therapeutic outcome) before re-implanting them in the patients, with the hope that there would be an effective antidepressant outcome from this intervention.

In conclusion, the alterations in reelin expression in both the CNS and periphery in depression (and in animal models of depression), the analysis of the functional roles of reelin (and dysfunctions in depression), and the observation of how reelin can rescue behavioral phenotypes in different paradigms, strongly suggest that systematically tackling the reelin system may be a good strategy for developing novel antidepressants. However, additional studies at multiple levels will be necessary to further this field and translate it to the clinic.

## Author Contributions

All authors have contributed to the acquisition of data, designing of the work, drafting and reviewing the manuscript, and gave the final approval to the version to be published. HJC and LEK have been involved in all the studies on which this manuscript is based, while KB, RR-T, MAM, and JB have been primarily involved in the hippocampal studies; and TR-B and JMO have been involved in the membrane protein clustering studies in depression patients.

## Funding

This work was funded by start-up funds from the College of Medicine and the College of Pharmacy and Nutrition at the University of Saskatchewan, a Saskatchewan Health Research Foundation Establishment Grant, and an NSERC-Discovery Grants to HJC; and by an NSERC- Discovery Grant to LEK.

## Conflict of Interest Statement

The authors declare that the research was conducted in the absence of any commercial or financial relationships that could be construed as a potential conflict of interest.
